# Statistical Design‐Guided Synthesis of Nanoarchitectonics of High‐Performance NiFeMoN Electrocatalyst through Facile One‐Step Magnetron Sputtering

**DOI:** 10.1002/advs.202308063

**Published:** 2024-01-28

**Authors:** Farid Attar, Astha Sharma, Bikesh Gupta, Siva Karuturi

**Affiliations:** ^1^ School of Engineering The Australian National University Canberra ACT 2601 Australia; ^2^ Department of Electronic Materials Engineering Research School of Physics The Australian National University Canberra ACT 2601 Australia

**Keywords:** electrochemical water splitting, magnetron sputtering, photoelectrochemical, statistical analysis, trimetallic

## Abstract

This study presents an innovative, statistically‐guided magnetron sputtering technique for creating nanoarchitectonics of high‐performing, NiFeMoN electrocatalysts for oxygen evolution reaction (OER) in water splitting. Using a central composite face‐centered (CCF) design, 13 experimental conditions are identified that enable precise optimization of synthesis parameters through response surface methodology (RSM), confirmed by analysis of variance (ANOVA). The statistical analysis highlighted a interaction between Mo% and N% in the nanostructured NiFeMoN and found optimizing values at 31.35% Mo and 47.12% N. The NiFeMoN catalyst demonstrated superior performance with a low overpotential of 216 mV at 10 mA cm^−2^ and remarkable stability over seven days, attributed to the modifications in electronic structure and the creation of new active sites through Mo and N additions. Furthermore, the NiFeMoN coating, when used as a protective layer for a Si photoanode in 1 m KOH, achieved an applied‐bias photon‐to‐current efficiency (ABPE) of 5.2%, maintaining stability for 76 h. These advancements underscore the profound potential of employing statistical design for optimizing synthesis parameters of intricate catalyst materials via magnetron sputtering, paving the way for accelerated advancements in water splitting technologies and also in other energy conversion systems, such as nitrogen reduction and CO_2_ conversion.

## Graphical abstract

The one‐step magnetron sputtering method is used to synthesize the nanoarchitectonics of trimetallic NiFeMoN catalysts, improving the Oxygen Evolution Reaction (OER). Statistical analysis identifies an interaction between Mo and N concentration, optimizing at 31.35% Mo and 47.12% N. The refined catalyst exhibits low overpotential (216 mV at 10 mA cm^‐2^) and impressive stability over seven days.

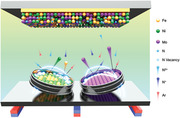

## 1 Introduction

The growing global energy demand, coupled with the urgent need to mitigate climate change, has propelled research on sustainable energy production. Green hydrogen, as an environmentally friendly energy carrier, has garnered significant attention as a promising candidate to address these challenges.^[^ ^1^ ^]^ In particular, electrochemical and photoelectrochemical (PEC) water splitting are emerging as favorable approaches for producing green hydrogen, through using abundant water resources and the limitless power of sunlight. However, the widespread adoption of these technologies is hampered by the lack of affordable, efficient, and industrially compatible electrodes for the oxygen evolution reaction (OER) and hydrogen evolution reaction (HER), two essential steps in the water‐splitting process.^[^ ^2^ ^]^

The OER is particularly critical, as it presents the bottleneck in the water splitting process due to its inherently sluggish kinetics and high overpotential. As a result, the efficiency of hydrogen production is directly influenced by the performance of OER catalysts. A major challenge in developing effective OER catalysts lies in the fact that many high‐performance catalysts reported thus far rely on noble metals, such as iridium or ruthenium. These materials are expensive and scarce, rendering them unsuitable for large‐scale applications.^[^ ^3^ ^]^ Consequently, recent research efforts have been focused on developing earth‐abundant catalysts, such as those based on nickel (Ni), iron (Fe), molybdenum (Mo), and cobalt (Co).^[^ ^4^ ^]^

Monometallic Ni‐based catalysts, although promising, suffer from an unsuitable electronic structure, which results in suboptimal OER catalytic performance.^[^ ^3^ ^]^ Bimetallic catalysts, such as NiFe, have been extensively studied in an attempt to address these shortcomings.^[^ ^5^ ^]^ However, some NiFe‐based catalysts still exhibit low catalytic activity and poor stability.^[^ ^6^ ^]^ To overcome these limitations, the incorporation of a third earth‐abundant metal, like Mo, has been proposed. Density functional theory (DFT) studies have demonstrated that introducing Mo and Fe into Ni(OH)_2_/NiOOH can enhance the stability and activity of the OER catalyst.^[^ ^7^ ^]^ Additionally, Mo can promote active Ni and Fe cations, improve charge redistribution, and better coordinate with OER intermediates, all of which contribute to improved OER performance.^[^ ^3^, ^8^, ^9^ ^]^

Furthermore, integrating nitrogen (N) atoms into the transition metal catalysts can significantly enhance their chemical, physical, and electronic properties, leading to improved conductivity and stability.^[^ ^1^, ^10^ ^]^ Consequently, we propose that introducing Mo and N into NiFe‐based catalysts presents a promising strategy for enhancing OER catalytic activity. However, fabricating effective complex catalysts often necessitates numerous synthesis steps and multiple experiments to determine the best performance. Therefore, employing a facile synthesis method and an appropriate optimization strategy to minimize the number of experimental tests is crucial for industrialization.

Traditional optimization methods, such as the “one‐variable‐at‐a‐time” (OVAT) approach, require a significant number of experiments, resulting in increased costs and time investment. Moreover, the OVAT approach overlooks the interactions of parameters, further limiting its utility.^[^ ^11^ ^]^ Alternatively, response surface methodology (RSM) is a statistical method that predicts a response by combining and optimizing parameters to achieve the highest response.^[^ ^12^ ^]^ RSM offers several advantages over the OVAT method, such as obtaining valuable information from minimal experiments, analyzing interaction effects, and modeling complex processes without requiring prior knowledge.^[^ ^13^ ^]^

In this context, magnetron sputtering, a physical vapor deposition technique, emerges as a highly potent method to fabricate our proposed trimetallic NiFeMoN catalysts for the OER. This technique is compatible with a wide range of substrates and has been extensively utilized for depositing mono‐ or multi‐metallic catalysts, capitalizing on the principles of nanoarchitectonics. Nanoarchitectonics is a methodology that involves the design and construction of functional materials from nanoscale units.^[^ ^14^ ^]^ The most significant advantages of magnetron sputtering over other physical and chemical deposition techniques include its production of high‐quality, adhesive, and stable films. It also offers cost efficiency, rapid processing, and consistent repeatability, which is particularly advantageous for large‐scale applications.^[^ ^15^ ^]^ Global market of magnetron sputtering is expected to reach 4.06 Bn in 2030 from US$ 2.51 Bn in 2021, further confirming its commercial viaibility.^[^ ^16^ ^]^ Recently, our group successfully demonstrated magnetron sputtering technique as a promising method to fabricate highly active and stable HER catalysts.^[^ ^17^ ^]^ No work to date has presented the magnetron sputtering method for fabricating trimetallic NiFeMo‐based catalyst for water splitting.

Herein, we leverage the magnetron sputtering method to develop nanoarchitectonics of trimetallic NiFeMoN catalysts via a one‐step process for the OER in alkaline solutions. In our approach, statistical analysis is employed to understand the individual and interactive effects of Mo and N concentration, which aids in optimizing the OER performance. We demonstrate that the incorporation of Mo and N into the NiFe catalyst enhances the OER performance by improving the electronic structure of Ni and Fe, and creating N vacancies and high‐valence Mo ions. The optimized NiFeMoN catalyst achieves superior performance with an overpotential of 216 mV at 10 mA cm^−2^ and excellent stability over seven days. Furthermore, we show the high performance of our optimized NiFeMoN catalyst when deposited on a silicon (Si) photoanode for PEC water splitting, exhibiting an applied‐bias photon‐to‐current efficiency (ABPE) of 5.2%. The sputtered NiFeMoN photoanode also demonstrates impressive operational stability, providing an effective chemical barrier against electrolyte corrosion. This study, therefore, provides a promising pathway for the fabrication of high‐performance, cost‐effective catalysts for sustainable green hydrogen production.

## 2 Experimental Section

### 2.1 Experimental Design and Statistical Model

The Response Surface Methodology (RSM) is a valuable tool for design and optimization, reducing the number of experimental tests required to achieve the desired results.^[^ ^18^ ^]^ The Central Composite Face‐centered (CCF) design is utilized for the experimental optimization of Mo% (A) and N% (N_2_:Ar flows in the sputtering chamber, B). Mo% and N% are identified as independent variables, with the overpotential (mV) at 10 mA cm^−2^ as the dependent variable. The CCF provides an accurate method for optimization, prediction, and understanding the impact of independent variables in complex processes.^[^ ^19^ ^]^

Table S1 (Supporting Information) summarizes the experimental ranges of Mo% and N%, obtained by preliminary experiments. The CCF design incorporates three levels, i.e., +1, 0, −1, representing higher levels, centre values, and lower levels, respectively. Figure S1 (Supporting Information) illustrates the CCF design, including the axial and factorial points of Mo% and N%. The axial points predict the curvature of the response surface, and the factorial points determine the interaction effects of the independent parameters.^[^ ^20^ ^]^

**Table** **1** summarizes 13 sets of experiments (containing 5 center points) conducted using CCF to develop the statistical model. With the application of CCF and RSM, a second‐order polynomial equation (Equation 1) is formulated to predict the overpotential at 10 mA cm^−2^ (Y, mV) as a function of Mo% (A, %) and N% (B, %).

(1) $$Y=\beta_{0}+\beta_{1}A+\beta_{2}B+\beta_{12}\mathrm{AB}+\beta_{11}A^{2}+\beta_{22}B^{2}$$

where β_0_ and β_12_ are interception and interaction coefficients, β_1_ and β_2_ represents the coefficients of the independent variables, and β_11_ and β_22_ are quadratic terms.

**Table 1:** The central composite face centered (CCF) design of experiment with predicted and actual values of OER overpotential at 10 mA cm^−2^.

Run	values of factors		OER OV [@10 mA cm^−2^, mV]	
	Mo% [A]	N% [B]	Actual	Predicted
1	0	0	293	290.3
2	80	0	290	292.7
3	0	70	254	253.7
4	80	70	276.5	281.6
5	0	35	250	252.8
6	80	35	276	268.1
7	40	0	269	268.9
8	40	70	250	245.1
9	40	35	235	237.8
10	40	35	234	237.8
11	40	35	238	237.8
12	40	35	244	237.8
13	40	35	233	237.8

Mean = 257.12 mV, Standard deviation = 21.4 mV

The data were presented as mean ± standard deviation (SD). The accuracy of the model and the influence of variables are assessed through an Analysis of Variance (ANOVA) examination. The *P* < 0.05 determines the statistical significance. RSM statistical model and optimization of the overpotential are conducted by Design‐Expert software (10.0.2.0 Stat‐Ease, Inc. Minneapolis, USA).

### 2.2 Catalyst Fabrication

Two layers of Ni foam, each 3 mm thick, were uniformly pressed together to produce a pressed Ni foam (PNF) with a thickness of 0.5 mm. The PNF was subsequently cleaned through sequential ultrasonication in acetone, ethanol, diluted HCl (3.0 m), and deionized water, each for a duration of 10 min. Trimetallic NiFeMoN catalysts were then deposited onto the PNF or Si photoanode using DC magnetron co‐sputtering (ATC 2400, AJA International Inc.). This process was conducted at a steady pressure of 4 mTorr, room temperature, and with a constant rotation of the substrate. NiFe (comprising 49.99% Ni and 49.99% Fe) and Mo (99.99%) targets were installed, and a flow of 20 sccm of argon gas was maintained in the sputter chamber throughout the process. The power of NiFe and Mo (each up to 150 W), along with the flow of N_2_, were adjusted according to the required Mo% and N% (as shown in Table 1) to achieve a film thickness of 100 nm. It is worth noting that in runs 1, 3, and 5 (Table 1), the Mo target was not ignited; only NiFe (49.99% Ni and 49.99% Fe) was deposited at a deposition power of 150 W.

A commercial p‐n type Si solar cell was procured to evaluate the photoelectrochemical (PEC) performance of the developed catalyst. The catalyst was deposited on the p‐type side of the silicon, with the silicon's light‐harvesting component being encapsulated by a combination of glass and epoxy. A titanium film was subsequently deposited to prepare the photoanode. This was accomplished by magnetron sputtering at a steady pressure of 4 mTorr and room temperature. A Ti target (99.99% purity) was installed, and a flow of 20 sccm of argon gas was maintained to achieve a titanium film thickness of 30 nm.

### 2.3 Material Characterization

A Si wafer coated with a SiO_x_ substrate was employed for various analyses, including scanning electron microscopy (SEM), scanning tunneling electron microscopy (STEM), X‐ray diffraction (XRD), and energy‐dispersive spectroscopy (EDS). Surface and cross‐sectional SEM (FEI Helios 600 NanoLab, accelerating voltage of 200 V‐30 kV) and STEM (JEOL JEM‐ARM200F) were conducted to examine the morphology of NiFe and NiFeMoN. XRD (PANalytical X'Pert PRO MRD) was performed using Cu Kα radiation (λ = 1.540598) within a scanning range of 2θ = 10 – 80° to determine the crystallinity. X‐ray photoelectron spectroscopy (XPS, Thermo ESCALAB250Xi), calibrated with a C 1s reference peak of 284.4 eV, was utilized to investigate the surface chemical properties of the samples.

### 2.4 Electrochemical and Photoelectrochemical Measurement

Electrochemical measurements are conducted in a three‐electrode configuration using a CHI660E potentiostat. A Pt plate and Ag/AgCl serve as the counter and reference electrodes, respectively. The distance between these three electrodes remains fixed for all electrochemical tests, with KOH (1 m, pH = 13) used as the electrolyte. The Nernst equation was employed to convert the Ag/AgCl potential to a reference hydrogen electrode (RHE) as follows:

(2) $$V_{\mathrm{RHE}}=V_{\mathrm{Ag}/\mathrm{AgCl}}+0.197+0.059\times pH_{\mathrm{electrolyte}}$$

For chronopotentiometry measurements, a saturated calomel electrode was used as the reference electrode due to its superior stability in alkaline electrolyte. Electrochemical impedance spectroscopy (EIS) measurements were undertaken at 1.47 V within a frequency range of 0.1 to 100 kHz. A non‐faradic region of 1.25 −1.35 V (RHE) was chosen to determine the double‐layer capacitance (Cdl) by increasing the scan rate from 20 to 100 mV s^−1^.^[^ ^21^ ^]^ Multi‐current chronopotentiometry was employed from 10 mA cm^−2^ to 100 mA cm^−2^, with increments of 30 mA cm^−2^ per 60 s (four steps) to examine charge transfer and side reactions. The amount of evolved O_2_ was measured by collecting gas samples at 30‐minute intervals and injecting them into a gas chromatograph (SHIMADZU GC‐2030 system, TCD, Argon carrier). Before conducting the electrochemical test at 10 mA cm^−2^, the system was thoroughly degassed using pure nitrogen to eliminate any trapped air. Photoelectrochemical measurements were performed under AM 1.5G illumination. The current‐voltage data from the photoelectrochemical measurement was converted to ABPE using the following equations:

(3) $$\;\eta_{ABPE}=\;\frac{\left(1.23-E\right)\times J\;\times \eta_{F}}{P}$$

where E represents the applied potential (RHE, V), η_F_ the faradic efficiency (considered 100%), J the photocurrent density (mA cm^−2^), and P the power of incident light (mW cm^−2^). Also, the above equation is known as a half cell efficiency.

## 3 Result and Discussion

### 3.1 NiFeMoN Synthesis by Co‐Sputter Deposition

**Figure 1:**
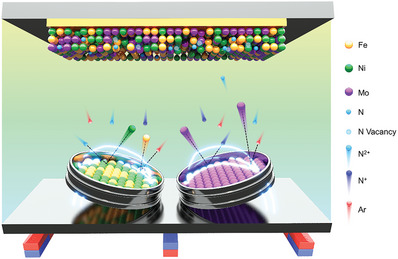
Schematic of NiFeMoN deposition by magnetron co‐sputtering method.

Rod‐shaped NiFeMoN films are synthesized at room temperature using magnetron co‐sputtering. **Figure** **1** illustrates a schematic of magnetron co‐sputtering, where NiFe and Mo targets are aligned in a confocal configuration toward the substrate. Once the desired vacuum is achieved, argon (serving as an inert gas) and nitrogen gases are introduced into the chamber. The magnetic fields, inherent in the targets, ionize argon atoms, facilitating the creation of a plasma region. Concurrently, N_2_ molecules undergo dissociation and/or ionization, following these reactions:

(4) $$e^{-}+N_{2}\;\to N_{2}^{+}+2e^{-}$$

(5) $$e^{-}+N_{2}\;\to N+N+e^{-}$$

(6) $$e^{-}+N_{2}\;\to N^{+}+N+2e^{-}$$

As a result of the bombardment by argon and nitrogen ions, Ni, Fe, and Mo particles are ejected from their respective targets. All sputtered particles, along with N ions, traverse from the plasma region to the substrates. The dissociated and ionized nitrogen reacts with the sputtered particles (Ni, Fe, and Mo), leading to the formation of the NiFeMoN film on the substrate. The process potentially generates N‐vacancies in the NiFeMoN film (Figure 1), analogous to the creation of N‐vacancies observed in sputter‐deposited Ni_3_N, Mo_2_N, MoN.^[^ ^17^, ^22^ ^]^ The presence and impact of N‐vacancies are investigated in the subsequent sections through various characterization tests. Detailed procedures of the magnetron sputtering deposition are described in the Materials and Methods section.

### 3.2 Statistical Analysis

#### 3.2.1 Statistical Model

The RSM model is leveraged to optimize the synthesis parameters of NiFeMoN with minimal experimentation. A CCF design is employed where Mo% and N% are specified as independent factors, and overpotential at 10 mA cm^−2^ is the dependent variable. Table 1 tabulates the OER results of thirteen experimental sets, realized through the CCF design, culminating in the development of the following second‐order polynomial equation:

(7) $$\begin{array}{ccc} Y & = & 290.346-1.102\;A-1.617\;B+0.004\;\mathrm{AB}\\ & & +0.015\;A^{2}+0.016\;B^{2} \end{array}$$

In this equation, Y, A, and B represent OER overpotential at 10 mA cm^−2^ (OV_10_, mV), Mo%, and N%, respectively. The statistical model (Equation 5) predicts the OER overpotential based on synthesis parameters within the selected ranges (Table 1). The values of OV_10_ predicted by the RSM model are compared with the experimental values in Figure S2a (Supporting Information). The distribution of points along the diagonal line in this figure underscores the impressive agreement between the statistical prediction and experimental data, evidencing high model accuracy. The normal probability of residuals (Figure S2b, Supporting Information) shows a well‐distributed set of residuals along the line, indicating no significant deviation from the assumptions underlying the analysis. The random scattering of residuals between horizontal lines (Figure S2c, Supporting Information) confirms the model's adequacy and reveals no discrepancy between predicted and experimental data.

Analysis of Variance (ANOVA) examination is conducted (Table S2, Supporting Information) to determine the model's accuracy and the impact of variables. A high model F‐value (33.36) and a low *p*‐value (<0.0001) demonstrate the model's accuracy with a 99% significance level.^[^ ^13a^ ^]^ Also, a low lack‐of‐fit p‐value for the model (2.39) affirms no notable correlation between lack‐of‐fit and pure error, as desired.^[^ ^12a^ ^]^ The coefficient of determination (R^2^), adjusted coefficient of determination (R^2^_adj_), and predicted determination coefficient are calculated as 0.96, 0.93, and 0.80, respectively, indicating that the experimental data align well with the predicted ones. The relative importance of variables can be examined via ANOVA: a smaller p‐value and a higher F‐value indicate greater relative importance. Therefore, Table S2 (Supporting Information) suggests that Mo% has a greater influence than N% on the OER water splitting performance, as the term corresponding to Mo% (A^2^) has the highest F‐value and the lowest P‐value.

#### 3.2.2 Individual and Interaction Effects

**Figure 2:**
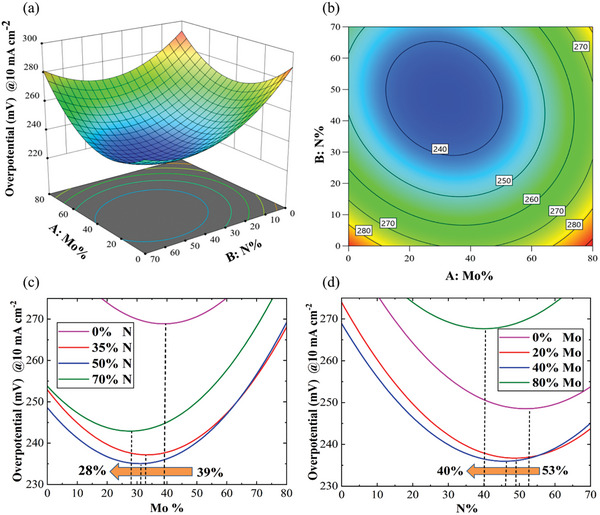
The response surface plots: a) 3D plot and b) contour plot of OER overpotential at 10 mA cm^−2^ as a function of Mo% and N%. c) Variation of optimized value of Mo% at different amounts of N%. d) Variation of optimized value of N% at different amounts of Mo%.

The statistical outcomes (Equation 5) are employed to produce 3D and contour response surface plots of OER OV_10_ as functions of Mo% and N%, depicted in **Figure** **2**a,b. It is evident that an excess of Mo and N negatively impacts the OER performance. However, by incorporating both Mo and N simultaneously, the OER performance can be enhanced. This implies a synergistic effect between Mo and N on OER performance, leading to reduced overpotential and more efficient OER.^[^ ^23^ ^]^ For a clearer understanding of the interaction effect, the individual impacts of Mo% and N% are presented in Figure S3 (Supporting Information), without considering the another variable (Mo% = 0 or N% = 0 in Equation 5). Figure S3a (Supporting Information) shows that increasing Mo% in the NiFe catalyst up to 39% decreases OER OV_10_, but beyond this point, OER OV_10_ begins to rise. Two other studies on NiFeMo catalyst synthesized by hydrothermal methods also achieved a similar percentage for OER water splitting.^[^ ^24^ ^]^ Figure S3b (Supporting Information) illustrates that OER OV_10_ reaches a minimum (≈53% of N) by increasing the N% pressure in the sputtering chamber. Nitrogen atoms combined with transition metals (such as Ni, Fe, and Mo) increase d‐electron density, align the electronic structure with noble metals, and can create nitrogen vacancies (NVs). However, at high N_2_ pressure during sputtering, NVs diminish, resulting in inferior water splitting performance.^[^ ^1^, ^10^ ^]^ Section 3.3 more distinctly examines the effects of Mo% and N% on OER water splitting through various characterization tests.

The 3D response plot (Figure 2a), the contour response plot (Figure 2b), and ANOVA result (F‐ and P‐values of AB in Table S2, Supporting Information) highlight the significant interaction effect of Mo% and N% on OER water splitting. To directly illustrate the interaction effect, simplified figures of the 3D plot are created by considering four different levels of N% (0%, 35%, 50%, and 70% in Figure 2c) and Mo% (0%, 20%, 40%, 80% in Figure 2d). Figure 2c,d display a substantial interdependency between the quantity of one factor and the optimum amount of the other. The optimum values of Mo% decrease from 39% to 28% with an increase in N% from 0% to 70% (Figure 2c), and the optimized points of N% drop from 53% to 40% by raising Mo% from 0% to 80% (Figure 2d). The presence of significant interdependency demonstrates that statistical analysis plays a crucial role to find the optimal percentages of Mo and N, where traditional optimization strategies, such as OVAT, may yield unrealistic results. Furthermore, Figure 2c,d indicate a lower sensitivity of optimum Mo% compared to N% by changing another variable, suggesting a greater effectiveness of Mo% on OER water splitting. This outcome aligns with the ANOVA results (Table S2, Supporting Information), where the F‐value of the Mo% quadratic term exceeds that of the N% quadratic term (66.54 > 22.55).

To achieve the best performance from NiFeMoN, the optimum values of Mo% and N% are determined using the numerical optimization method of Design‐Expert. The model predicts that the minimum OER OV_10_ of 229.56 mV can be achieved under the optimum values of 31.35 Mo% and 47.12 N%. To verify the accuracy of this predicted model, OER water splitting measurements are carried out on three different samples under the optimum conditions. The average OER OV_10_ is found to be 227 ± 3 mV, which is close to the proposed value. This suggests that the proposed statistical model is accurate in optimizing the synthesis parameters of magnetron sputtering. In subsequent sections, the optimized point of NiFeMoN (Mo% = 31.35 and N% = 47.12) is identified and denoted as Opt NiFeMoN.

### 3.3 Characterisation of NiFeMoN Electrocatalyst

**Figure 3:**
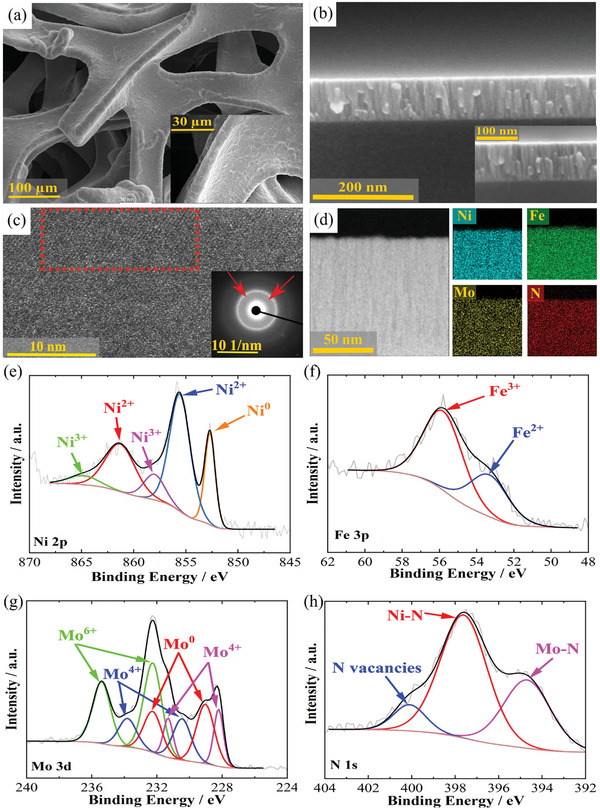
a) SEM images of Opt NiFeMoN from top view and b) cross‐sectional view. c) STEM image of Opt NiFeMoN with selected area electron diffraction pattern (inset). d) STEM cross‐sectional image and corresponding elemental mapping of Opt NiFeMoN. XPS spectra e) Ni 2p, f) Fe 3p, g) Mo3d, and h) N 1s in Opt NiFeMoN.

A one‐step magnetron co‐sputtering method is employed to deposit earth‐abundant trimetallic NiFeMoN. The SEM image (**Figure** **3**a) illustrates a uniform and compact film of Opt NiFeMoN on the 3D porous PNF. Comparing the SEM images of Opt NiFeMoN (Figure 3a), NiFe (Figure S4a, Supporting Information), and bare PNF (Figure S4b, Supporting Information) reveals no significant morphological change, indicating that magnetron sputtering deposits compact films on PNF without aggregation. Also, the nanoparticle morphology of the catalyst on SiO_x_/Si without any cracks (the average feature size is 9.5 nm) confirms that sputtered NiFeMoN films can potentially serve as a protection layer for Si photoelectrode (Figure S4c, Supporting Information). The cross‐sectional SEM image (Figure 3b) shows 100 nm‐thick vertical rod‐shaped nanostructures of Opt NiFeMoN. This rod‐shaped morphology can increase the catalytic surface area, reactant adsorption, and overall performance.^[^ ^17b^ ^]^

The STEM image and selected area electron diffraction (SAED) demonstrate the amorphous quality of Opt NiFeMoN with poor crystallinity (Figure 3c). Additionally, no extra peaks are present in the XRD analysis of Opt NiFeMoN (Figure S5a, Supporting Information) compared with bare PNF, which exhibits a polycrystalline face‐centered‐cubic (fcc) Ni structure. The XRD patterns of Opt NiFeMoN deposited on SiO_x_/Si are analyzed, revealing a broad peak due to poorly crystalline metallic Ni (Figure S5b, Supporting Information).^[^ ^3^ ^]^ The cross‐section (Figure 3d; Figure S6a, Supporting Information) and top‐section (Figure S6b, Supporting Information) EDS elemental mappings of Opt NiFeMoN highlight the uniform distribution of Ni, Fe, Mo, and N elements. It also shows the Mo/Ni/Fe/N ratio is 1:1.15:1.03:0.33, aligning well with the optimum sample composition.

The composition of Opt NiFeMoN is examined by X‐Ray photoelectron spectroscopy (XPS), confirming the presence of Ni, Fe, Mo, and N elements (Figure 3e–h). The Ni 2p spectrum (Figure 3e) deconvolves into Ni^0^ (852.1 eV), Ni^2+^ (861.4 eV and 855.1 eV), and Ni^3+^ (864.94 eV and 858.1 eV).^[^ ^3^, ^5^, ^25^ ^]^ The Fe 3p (Figure 3f) and Fe 2p (Figure S7, Supporting Information) spectra correspond to Fe^2+^ (53.4 eV and 710.75 eV)^[^ ^3^, ^26^ ^]^ and Fe^3+^ (55.8 eV and 707.9 eV).^[^ ^26^, ^27^ ^]^ From the XPS Mo 3d spectra (Figure 3g), six sub‐peaks related to Mo^0^ (233.3 eV and 229.1 eV),^[^ ^28^ ^]^ Mo^+4^ (233.8 eV and 230.4 eV),^[^ ^25^ ^]^ and Mo^6+^ (235.4 eV and 232.3 eV) ^[^ ^8^, ^24^ ^]^ are detected. It is concluded that the presence of high‐valence molybdenum ions (Mo^+4^ and Mo^6+^) significantly enhances the OER performance.^[^ ^9^ ^]^

Comparing Ni 2p of Opt NiFeMoN (Figure 3a) and NiFe (Figure S8a, Supporting Information) supports that Ni^0^ increases via Mo and N addition. It was realized the synergistic effects of Ni^0^ and Mo^4+^ on the OER improvement, as Ni° facilitates oxygen intermediates adsorption, and Mo^4+^ pulls the electrons of adsorbed oxygen intermediates. Comparing Fe 2p and Fe 3p spectra of NiFe and NiFeMoN (Figures S7 and S8b, Supporting Information; Figure 3f) indicates the Fe^3+^ and Fe^2+^ formations by incorporating the Mo ions resulting in improving OER performance.^[^ ^8^ ^]^ Also, moving to lower binding energy of Fe^2+^ after Mo addition is depicted in Figure S7 (Supporting Information) which can to be a result of increasing Fe^2+^.^[^ ^3^ ^]^ The N 1s spectra of Opt NiFeMoN reveal the peaks at 394.6 eV, 397.66 eV, and 400.1 eV which are related to Ni‐N, Mo‐N and N vacancies (Figure 3h).^[^ ^17^, ^29^ ^]^ It was found that Ni‐N improves wettability, Mo‐N boosts catalytic activity and N vacancies serve as a new active site and increase stability.^[^ ^17^, ^30^ ^]^

### 3.4 Electrochemical Performance of Optimized NiFeMoN

**Figure 4:**
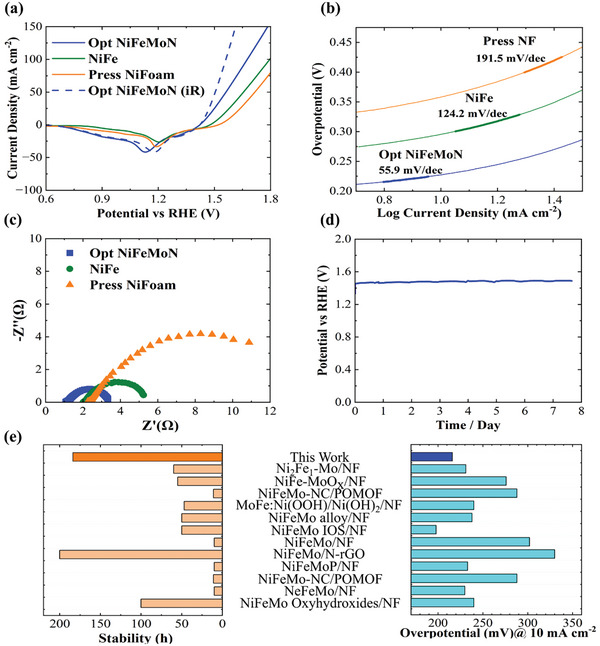
Comparison of Opt NiFeMoN, NiFe and PNF: a) Linear sweep voltammetry (LSV) curves (with 100% and without iR correction) in 1 m KOH, b) Tafel plots, c) Nyquest plot. d) Chronopotentiometric measurement of Opt NiFeMoN at 227 mV of overpotential, e) OV_10_ and stability comparisons of Opt NiFeMoN with previously reported NiFeMo‐based electrocatalysts.

The OER electrochemical performance of Opt NiFeMoN is examined in a KOH solution (1 m, pH 13) in a three‐electrode cell at room temperature. The linear sweep voltammetry (LSV, **Figure** **4**a) of Opt NiFeMoN/PNF presents a more desirable performance (overpotential of 227 mV at 10 mA cm^−2^ without iR correction and 216 with 100% iR correction) than NiFe/PNF (293 mV) and bare PNF (349 mV). Tafel plots are shown in Figure 4b, drawn from the corresponding LSV, to further explore the catalytic activity and inherent kinetics of NiFeMoN. A much lower slope of Opt NiFeMoN (55.9 mV dec^−1^) than NiFe (124.2 mV dec^−1^) and bare PNF (191.5 mV dec^−1^) indicates that Mo and N additions can reduce energy consumption, improve energy conversion efficiency, and increase the O_2_ generation rate by changing the OER mechanism. ^50^ Based on the Tafel slope values, NiFe (124.2 mV dec^−1^) follows a metal oxyhydroxide pathway by transferring two electrons; however, some active sites in NiFeMoN (55.9 mV dec^−1^) follow both oxide and oxyhydroxide pathways by transferring three electrons (Figure S9, Supporting Information). Thus, changing the two‐electron pathway to three‐electron by adding Mo and N increases the energy conversion yield and the oxygen generation rate.

Electrochemical impedance spectroscopy (EIS) measurements are conducted to further investigate the performance of the catalyst by analysing the series and charge transfer resistances (Figure 4c). The lower series resistance of Opt NiFeMoN, corresponding to the radius of the semicircle, can result from the generation of Mo^4+^ and metal nitrides (Mo‐N and Ni‐N) (Figure 3g,h), and the improvement of electrical conductivity.^[^ ^23^, ^31^ ^]^ Also, N vacancies and the development of Fe^3+^ can enhance charge transfer by gathering excess electrons and accelerating the oxidation‐reduction of Fe^2+^ and Fe^3+^ during OER, respectively.^[^ ^32^ ^]^

The electrochemical surface area (ECSA) of NiFeMoN and NiFe are obtained by double‐layer capacitance measurement. The double‐layer capacitance (C_dl_) is found by the cyclic voltammetry (CV) in the non‐faradic area at different scan rates (Figure S10, Supporting Information). Adding Mo and N increase C_dl_ (from 2.36 mF cm^−2^ of NiFe to 4.67 mF cm^−2^ of NiFeMoN) and ECSA, which results in higher OER catalytic activity. To examine the occurrence of side reactions, multi‐current chronopotentiometry of Opt NiFeMoN is performed by increasing the current density from 10 mA cm^−2^ to 100 mA cm^−2^ with a rise of 30 mA cm^−2^ per 60 s (Figure S11, Supporting Information). The measured potential at different current densities is stable without visible fluctuation, specifying quick significant mass and charge transfers without side reactions.^[^ ^33^ ^]^ The measured faradaic efficiency of Opt NiFeMoN aligns with the calculated O_2_ gas under 227 mV overpotential, indicating that most of the consumed charges contribute to the OER reaction without any side reactions (Figure S12, Supporting Information).

The chronopotentiometric measurement (Figure 4d) indicates the long‐term stability of the Opt NiFeMoN catalyst at 10 mA cm^−2^ for more than 7 days (183 h). The overpotential and stability of Opt NiFeMoN are further compared with other NiFeMo‐based catalysts in the literature (Figure 4e; Table S3, Supporting Information). Opt NiFeMoN reveals both high catalytic performance and stability, which are essential for industries. These results verify that our facile and fast processing method can develop effective electrodes for future water‐splitting industries. Additionally, the RSM statistical model can be employed for the synthesis of complex water‐splitting catalysts to obtain the best outcome with minimal experimental tests. This method can be extended to catalyst development for fuel cells, batteries, and other energy conversion systems such as CO_2_ conversion and N_2_ fixation.

Magnetron sputtering offers a simple one‐step deposition process for NiFeMoN catalysts. Material characterisation analyses demonstrate the capability of this technique to deposit a thin catalyst layer (Figure 3b), thereby enhancing water splitting performance and catalytic conductivity. The well‐distributed elements and the formation of rod‐shaped nanostructures achieved through sputtering (Figure 3d,b) further contribute to the improved efficiency of water splitting.

A key distinction of magnetron sputtering is the generation of metallic elements (Ni^0^ and Mo^0^ in Figure 3e,g). This stands in contrast to NiFeMo‐based catalysts produced by alternative deposition methods such as supersaturated coprecipitation, electrodeposition, and inverse‐opal techniques, where metallic elements are notably absent.^[^ ^8^, ^34^ ^]^ The presence of metallic elements is significant due to their inherent catalytic activity, which enhances catalytic conductivity and improves the overall mechanism.^[^ ^9^, ^24^ ^]^

The OER mechanism of NiFeMoN can be explained through Tafel slopes (Figure S9, Supporting Information) with two potential pathways: the oxides and the oxyhydroxide. In the oxides pathway, involving a 4‐electron process, simultaneous oxidation of coordinated hydroxide and the formation of O‐O bonds occur, leading to immediate oxygen generation through the elimination and self‐reduction of catalytic sites. The oxyhydroxide pathway could involve the below reaction leading to oxygen generation, where M can be Ni, Fe, and Mo (Figure S13, Supporting Information)^[^ ^32a^ ^]^:

(8) $$M^{2+}-OH+\;OH^{-}\left(aq\right)\to M^{3+}\left(O\right)-OH+H^{+}\left(aq\right)+e^{-}$$

(9) $$\begin{array}{ccc} M^{3+}\left(O\right) & - & OH+\;OH^{-}\left(aq\right)\to M^{2+}-OH+H^{+}\left(aq\right)\\ & + & O_{2}\left(g\right)+e^{-} \end{array}$$

### 3.5 Photoelectrochemical Performance of Opt NiFeMoN with Si Photoanode

Si is a promising, low‐cost, earth‐abundant, and manufacturing‐friendly semiconductor that effectively absorbs light and transfers charge carriers. However, it suffers from corrosion in harsh alkaline electrolytes, leading to poor stability.^[^ ^35^ ^]^ Sputtered Opt NiFeMoN can chemically protect the Si/SO_x_ from corrosion by generating a compact film (Figure S4, Supporting Information). To demonstrate the applicability of magnetron sputtering on PEC water splitting, sputtered Opt NiFeMoN is investigated as a stabilizing co‐catalyst for a Si photoanode. A decoupled photoanode design is utilized wherein the irradiated and electrocatalytic components are physically separated.^[^ ^5b^ ^]^ This design improves PEC performance by reducing photocurrent losses and increases cell lifetime.^[^ ^36^ ^]^ Also, a non‐transparent catalyst can be optimized and used without considering the photoanodic components.

**Figure 5:**
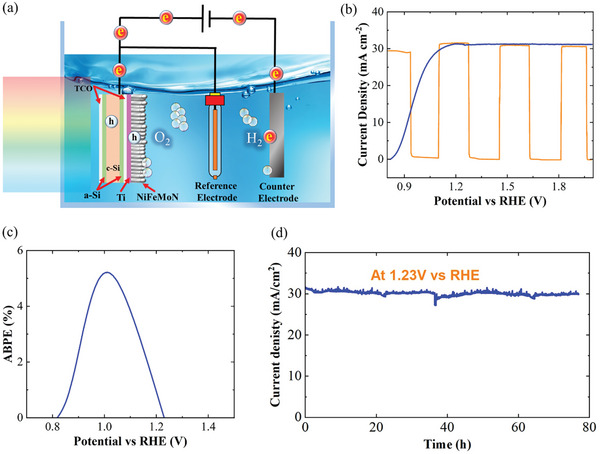
a) Schematic of PEC water splitting using sputtered NiFeMoN Si photoanode, b) linear sweep voltammetry (LSV) (Blue) and light on‐off irradiation (orange) of NiFeMoN photoanode, c) corresponding applied‐bias photon‐to current efficiency (ABPE), d) chronoamperometric measurement at 1.23 V versus RHE in 1 m KOH under AM1.5G illumination.

To prepare a Si photoanode, 30 nm of Ti and 100 nm of Opt NiFeMoN are sequentially deposited on the Si sample by magnetron sputtering. A thin Ti film improves adhesion and charge carrier transfer.^[^ ^17b^ ^]^ The light‐harvesting component of Si is encapsulated by glass to chemically protect the Si surface. **Figure** **5**a reveals the decoupled PEC schematic and the water splitting mechanism of the NiFeMoN/Ti/Si photoanode. The electron holes (charge carriers) are generated by AM1.5G illumination on the front side of the photoanode where the irradiated energy is higher than the Si bandgap. The generated holes move to the rear side of the photoanode and then to the rod‐shaped NiFeMoN, participating in the oxygen evolution reaction.

Figure 5b demonstrates the LSV photo‐response of the NiFeMoN‐coated Si photoanode in a three‐electrode configuration. The saturation current density is 31 mA cm^−2^, which falls within the expected range of the Si photoelectrode. The device quickly responds to the measured potential due to the measured photocurrent under light‐off conditions (Figure 5b). The applied‐bias photon‐to‐current efficiency (ABPE) was found to be 5.3% at 1.00 V versus RHE, calculated by the LSV curve (Figure 5c). The ABPE value obtained by the Si photoanode is remarkable, compared with other photoanodes in a KOH electrolyte (1 m),^[^ ^35^, ^37^ ^]^ highlighting the effectiveness of the NiFeMoN as a cocatalyst.

Furthermore, a chronoamperometric measurement (J–t) is conducted at 1.23 V to determine the photoanode's stability under AM1.5G illumination. Figure 5d illustrates that the photocurrent remains stable after 76 h, and the LSV curve is nearly unchanged after the stability test (Figure S14, Supporting Information). These results show that sputter‐deposited NiFeMoN chemically protects Si as a passivation layer in a photoanode device. The performance of NiFeMoN developed in this work is compared with recent Si photoanode studies (both coupled and decoupled conditions) in 1 m KOH (Table S4, Supporting Information). It confirms that the photoanode device developed by a one‐step magnetron sputtering process and optimized by a statistical model (this work) indeed leads to a remarkable performance.

## 4 Conclusion

We successfully developed a one‐step magnetron sputtering method to synthesise nanoarchitectonics of NiFeMoN catalysts for water splitting. This process utilizes a Central Composite Face‐centered (CCF) design and Response Surface Methodology (RSM) for efficient statistical modelling and optimization. This approach, validated by ANOVA, emphasizes the crucial interdependence between Mo% and N%, with the optimized values of 31.35% and 47.12% respectively, enhancing oxygen evolution reaction (OER) performance and stability (up to 183 h in alkaline electrolyte). The introduction of Mo and N into NiFe catalysts improves the electronic structure, electrochemical surface area, and OER kinetics while creating N vacancies and modifying valence of Mo ions. This results in a catalyst that not only demonstrates superior OER performance but also effectively protects Si photoanodes from photo‐corrosion in alkaline conditions, achieving 5.2% efficiency with 76 h of stability. Due to its elevated electronic structure, modified Mo valence, and the creation of N vacancies, this catalyst holds the potential for electrocatalytic nitrogen reduction. Furthermore, the magnetron sputtering method can be extended to catalyst development for fuel cells, batteries, and other energy conversion systems, including CO_2_ conversion. Additionally, the application of a statistical model in this field can minimize the requirement for extensive experimentation, leading to enhanced performance.

## Conflict of Interest

The authors declare no conflict of interest.

## Supporting information

Supporting Information

## Data Availability

The data that support the findings of this study are available from the corresponding author upon reasonable request.
