# Supplementary material for: Statistical Design‐Guided Synthesis of Nanoarchitectonics of High‐Performance NiFeMoN Electrocatalyst through Facile One‐Step Magnetron Sputtering
Source: Adv Sci (Weinh). 2024 Jan 28;11(14):2308063. doi: 10.1002/advs.202308063 (PMC11005699; doi:10.1002/advs.202308063)
Supplement: Supplementary file 1 — Supporting Information [file ADVS-11-2308063-s001.pdf]

ADVANCED SCIENCE
Open Access

Supporting Information

for *Adv. Sci.*, DOI 10.1002/advs.202308063

Statistical Design-Guided Synthesis of Nanoarchitectonics of High-Performance NiFeMoN Electrocatalyst through Facile One-Step Magnetron Sputtering

Farid Attar, Astha Sharma, Bikesh Gupta and Siva Karuturi**

Statistical Design-Guided Synthesis of Nanoarchitectonics of High-Performance NiFeMoN Electrocatalyst through Facile One-Step Magnetron Sputtering

Farid Attar,[1] Astha Sharma,[1*] Bikesh Gupta,[2] Siva Karuturi,[1*]

[1] School of Engineering, The Australian National University, Canberra, Australian Capital Territory 2601, Australia

[2] Department of Electronic Materials Engineering, Research School of Physics, The Australian National University, Canberra, ACT 2601, Australia

Email: siva.karuturi@anu.edu.au, astha.sharma@anu.edu.au

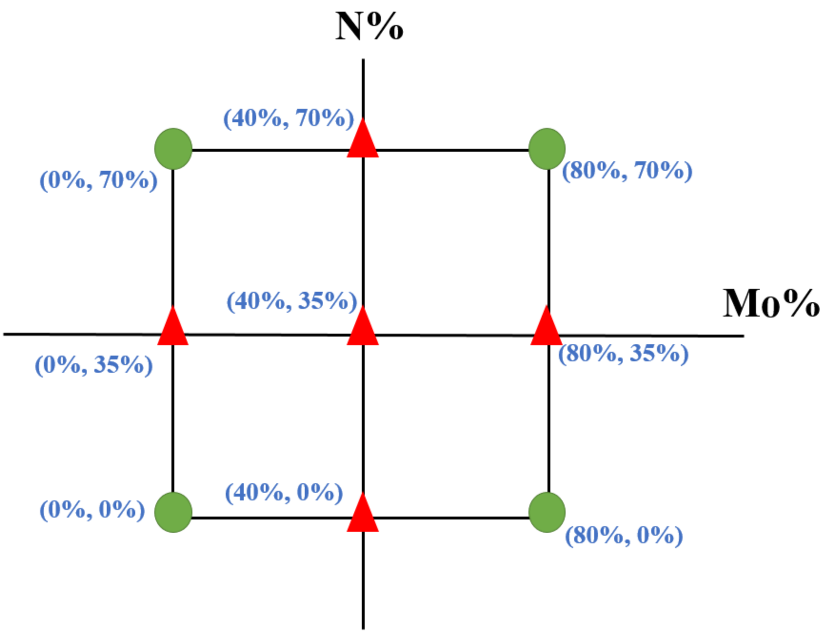

Figure S1. The central composite face centered (CCF) design of experiment for Mo% and N% including the factorial points (blue circles) and axial points (red triangles).

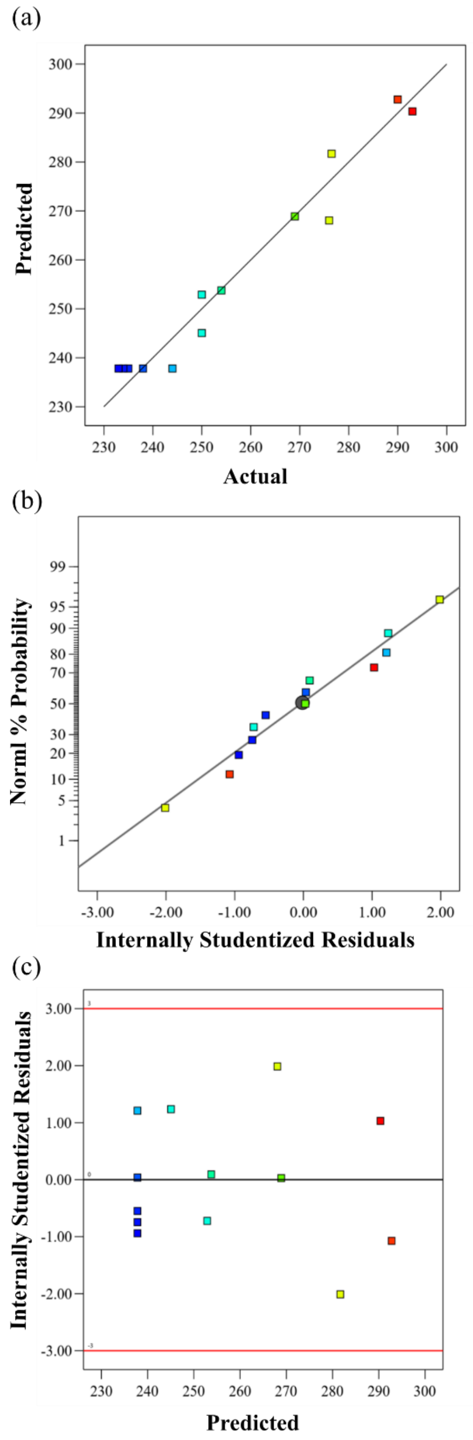

Figure S2. Plot of the actual and predicted values of overpotential at 10 mA cm^{-2} (a); The normal probability of residual for 13 set of tests (b); Plot of the residual versus model predictions for the overpotential at 10 mA cm^{-2} (c).

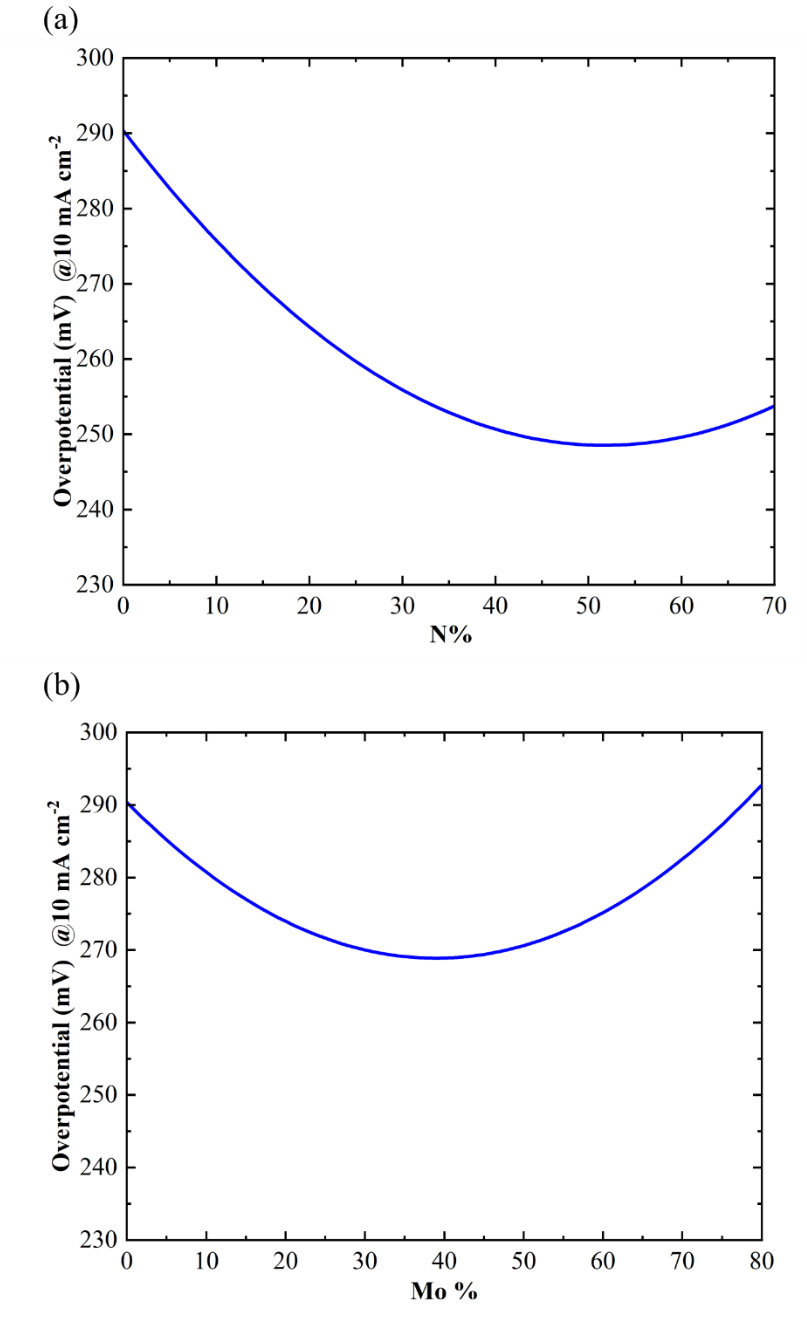

Figure S3. Individual effect of N% (a) and Mo% (b) on OER overpotential at 10 mA/cm^2.

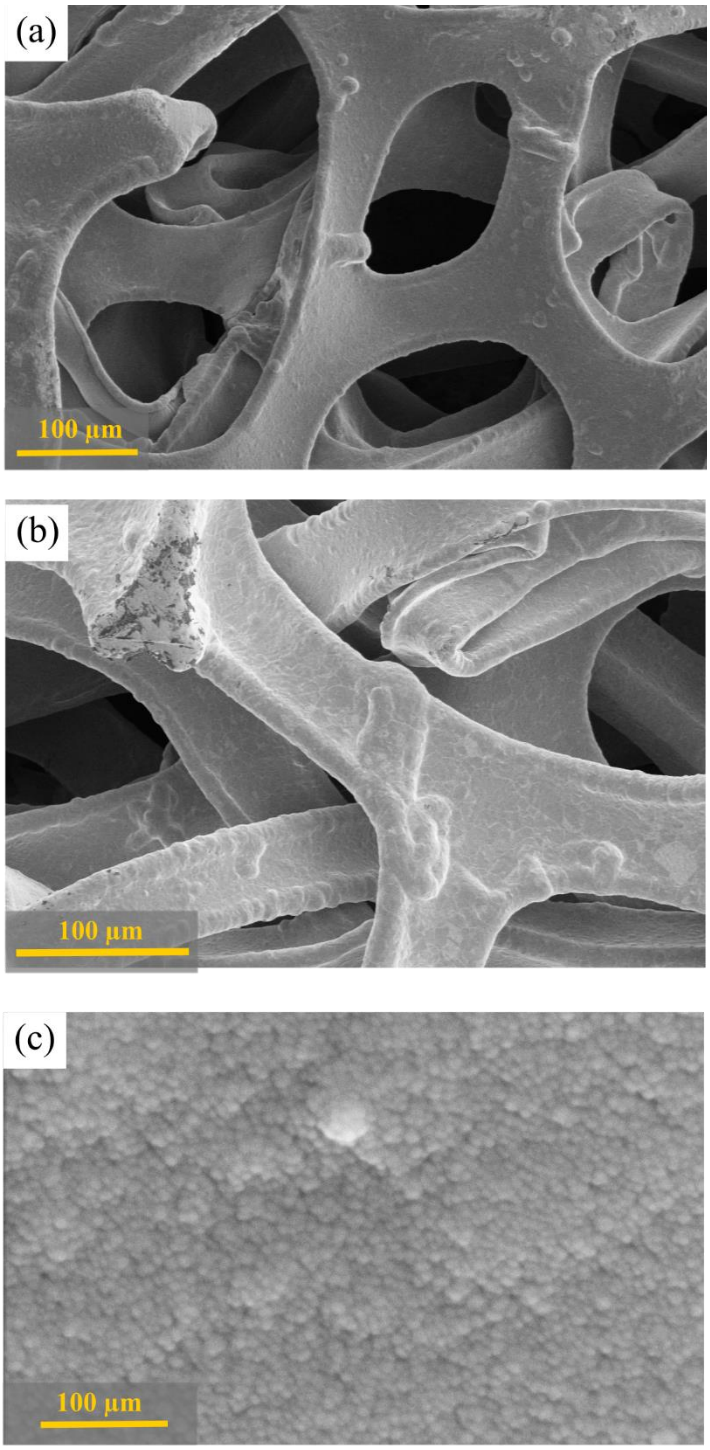

Figure S4. SEM images of (a) NiFe deposited on PNF and(b) bare PNF, (c)Opt NiFeMoN on SiO_x/Si.

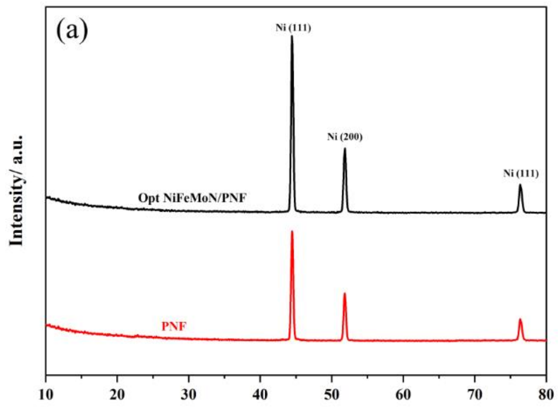

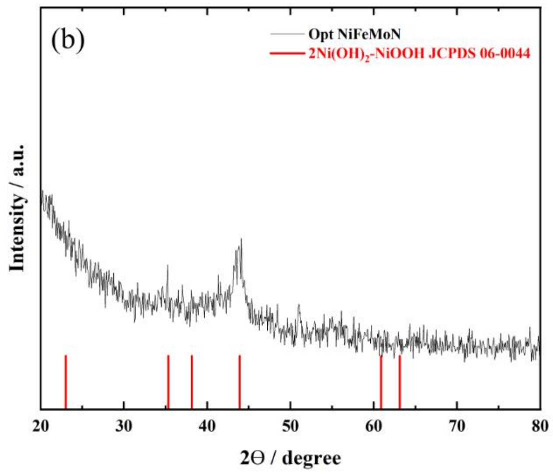

Figure S5. XRD patterns of Opt NiFeMoN deposited on PNF and bare PNF (a), XRD pattern of NiFeMoN deposited on SiO_x/Si.

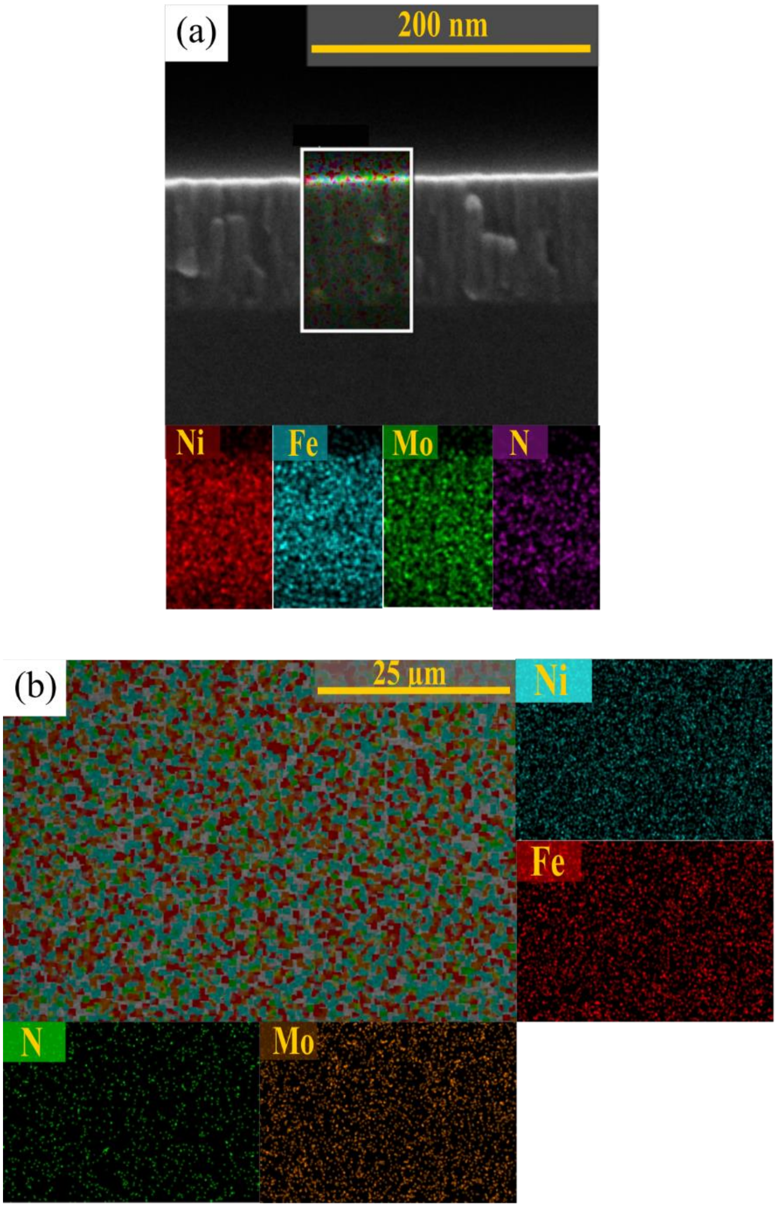

Figure S6. EDS mapping of Opt NiFeMoN from cross-section view (a) and top view (b).

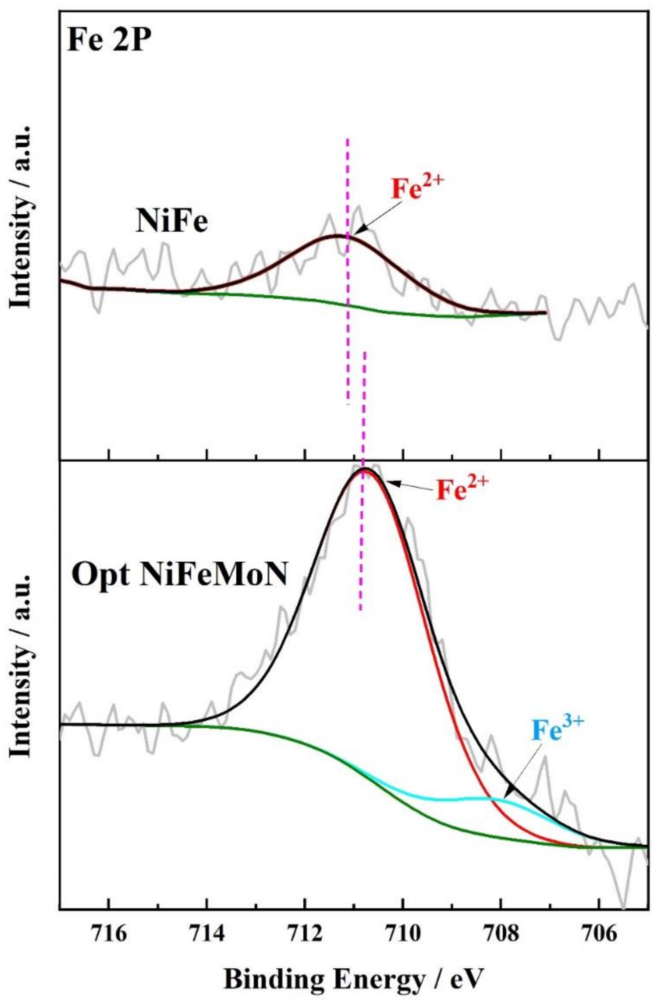

Figure S7. XPS spectra of Fe 2p for NiFe and Opt NiFeMoN.

(a)

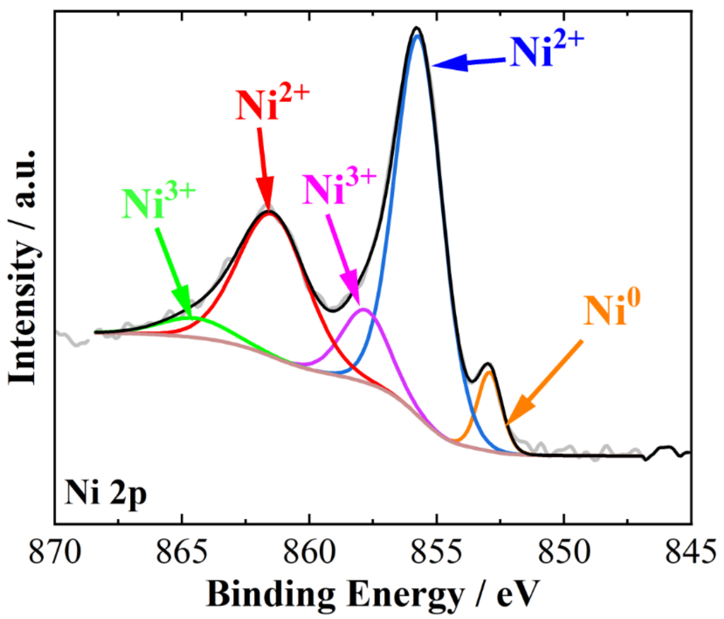

(b)

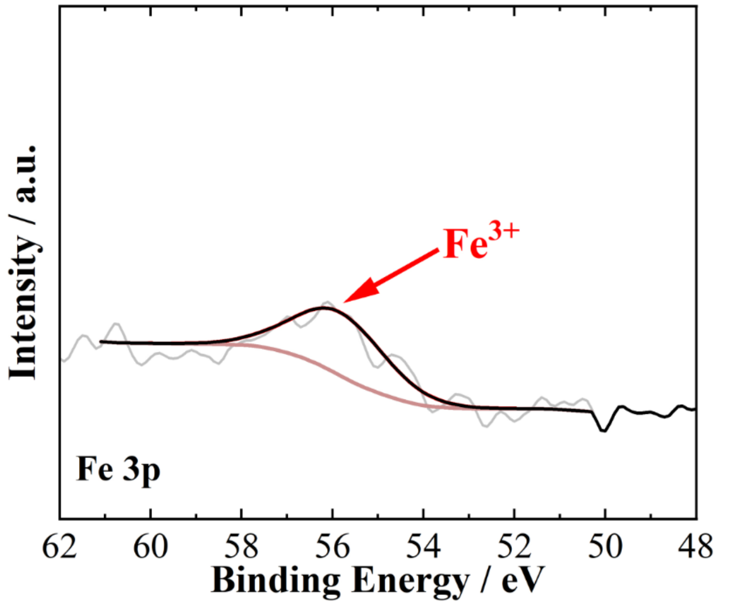

Figure S8. XPS spectra of Ni 2p and Fe 3p for NiFe.

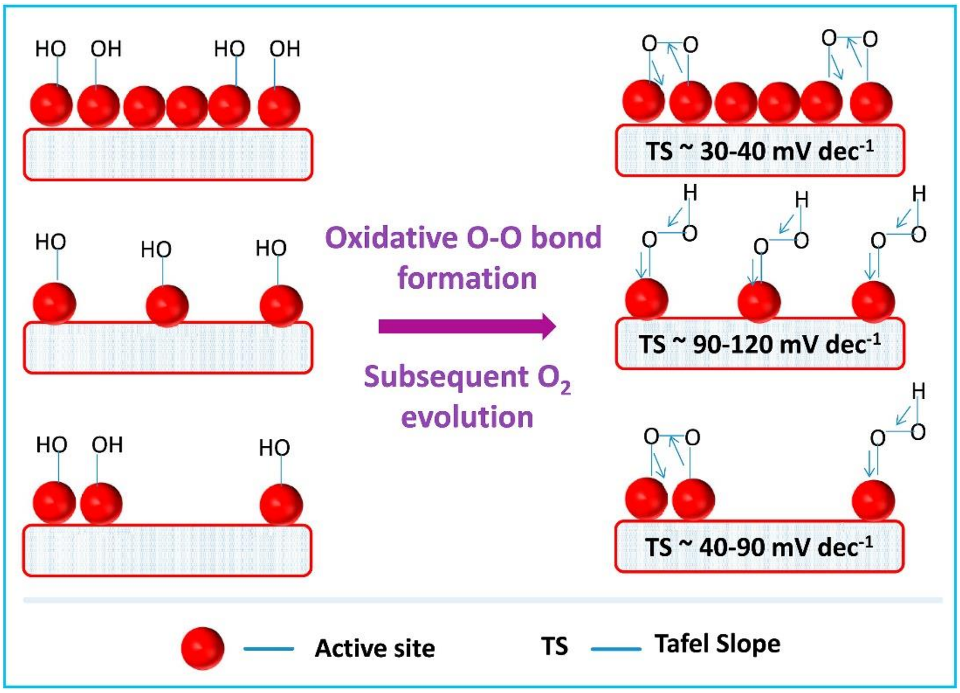

Figure S9. Schematic diagram of different OER mechanisms related with Tafel slopes. Reproduced from ref [1]*Copyright 2017 Elsevier publishing.*

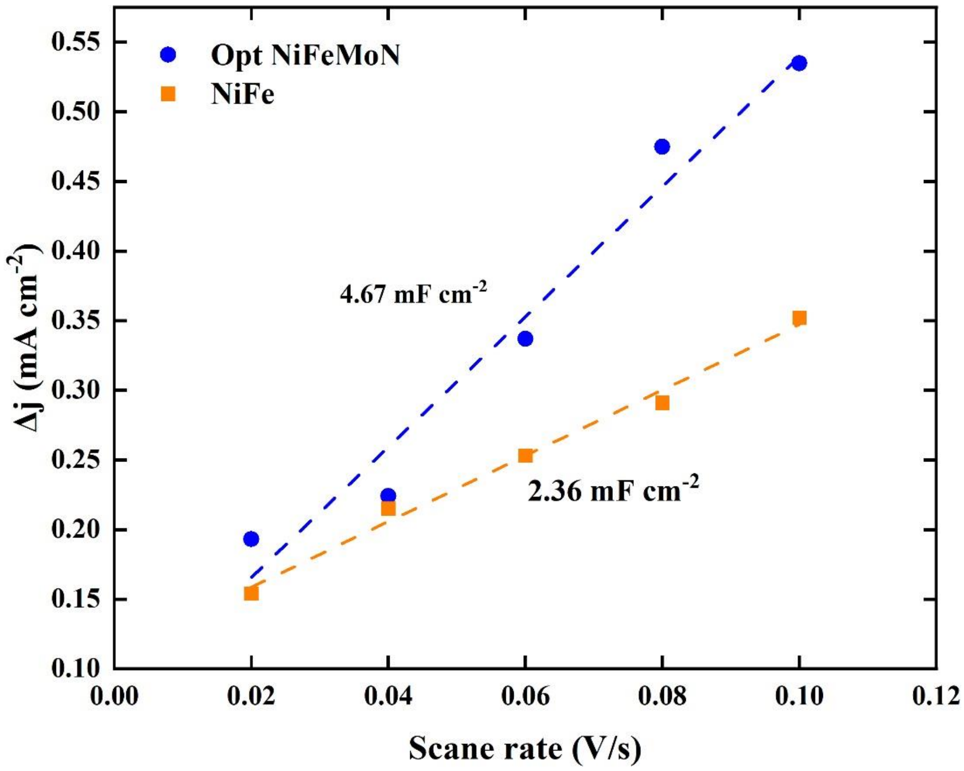

Figure S10. The linear fitting of double layer capacitances of Opt NiFeMoN and NiFe.

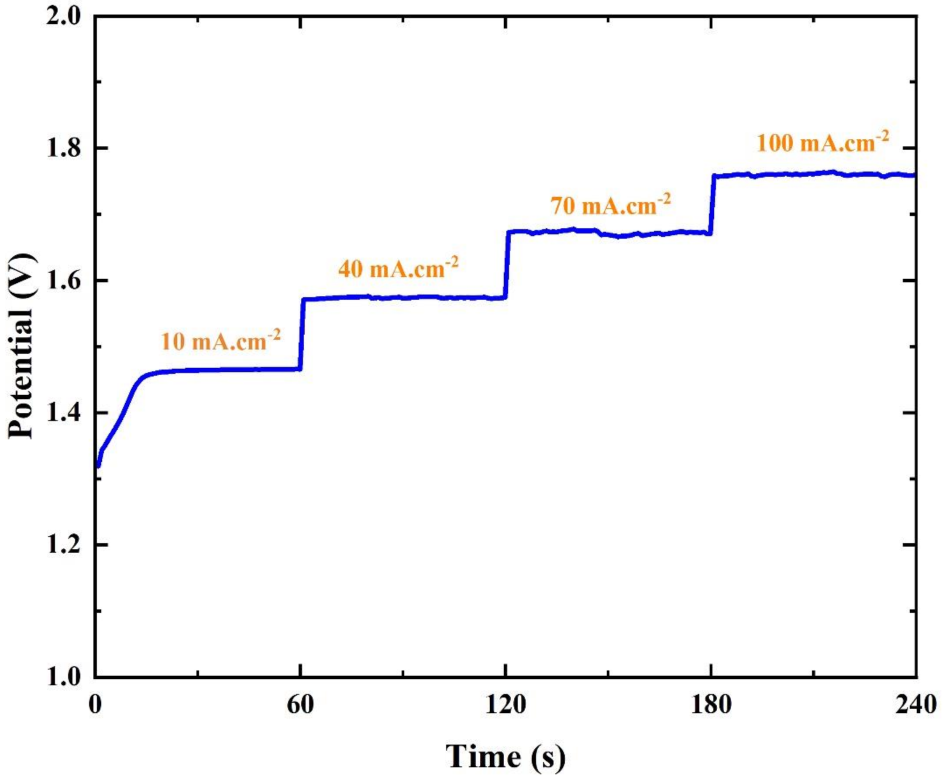

Figure S11. Consecutive multi-step chronopotentiometry of NiFeMoN.

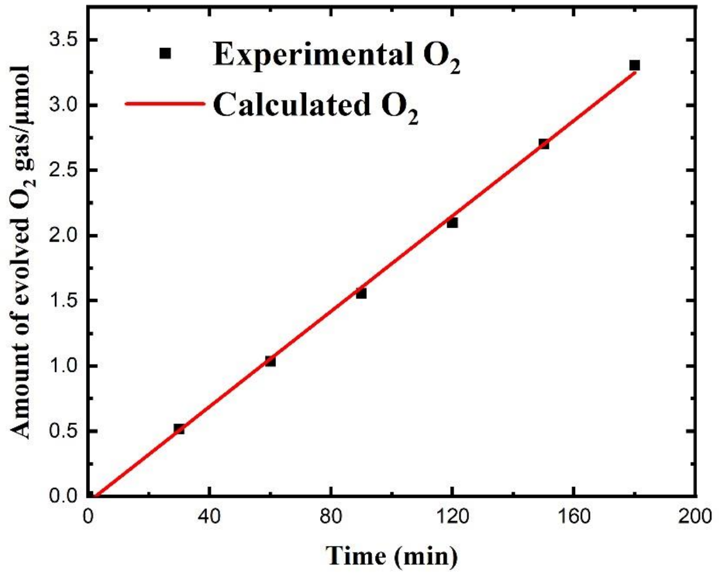

Figure S12. The measured O_2 detected by gas chromatography and the calculated O_2 amount of Opt NiFeMoN

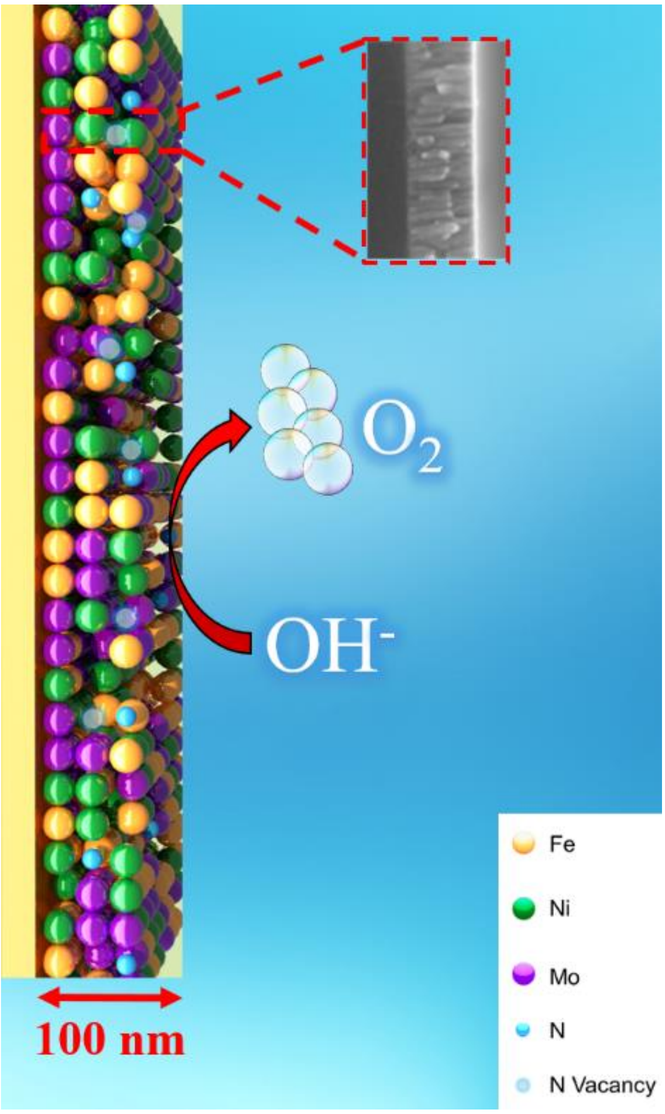

Figure S13. Schematic of PEC water splitting using sputtered NiFeMoN.

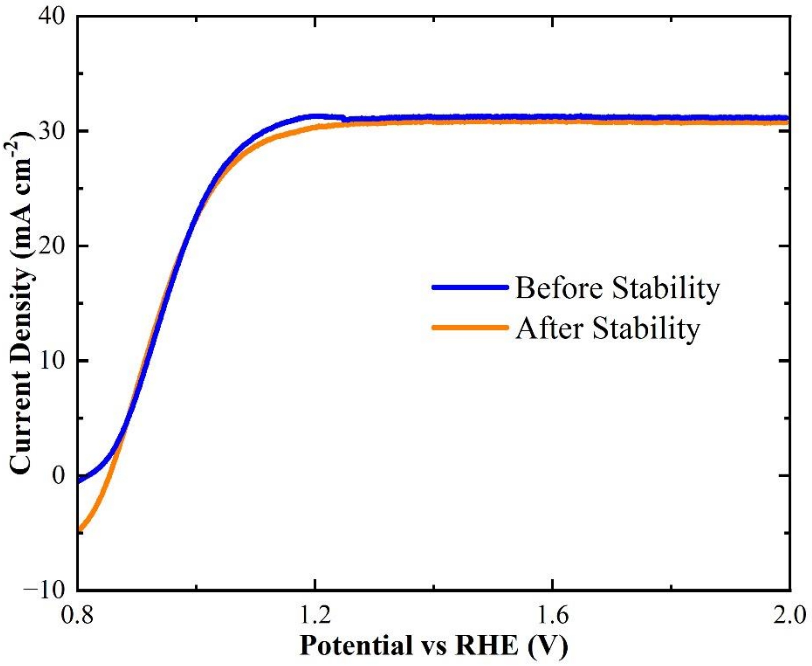

Figure S14. J-V curves of photoanode before and after the stability test.

Table S1. Experimental levels and ranges of Mo% and N% for OER water splitting.

Factors	Ranges and Levels		
	-1	0	+1
Mo (A, %)	0	40	80
N (B, %)	0	35	70

Table S2 Analysis of variance for the quadratic model of OER overpotential at 10 mA/cm^2.

Source	*F*-value	*p*-value Prob>*F*
Model	33.36	< 0.0001
A	10.91	0.0131
B	26.95	0.0013

AB	15.14	0.0177
A^2	44.86	< 0.0001
B^2	32.08	0.0021
Lack of Fit	2.39	0.2072

R^2 =0.960; Adjusted R^2=0.931; Predicted R^2=0.802; Mean= 257.12, Standard deviation=5.62.

A: Mo %; B: N%.

Table S3. Comparison of OER activity of Opt NiFeMoN with previously reported NiFeMo-based electrocatalysts.

Catalysts	Substrate	overpotential V vs. RHE at 10 mA cm^{-2}	Stability (h)	Reference
**NiFeMoN**	**Ni foam**	**216**	**183**	**This work**
NiFeMo	N-rGO	330	200	[2]
NiFeMo oxyhydroxidest	Ni foam	240	100	[3]
NiFeMo	Ni foam	230	10	[4]
NiFeMo-NC	POMOF (polyoxometalate based metal-organic framework)	288	11	[5]
NiFeMoP	Ni foam	233	10	[6]
NiFeMo	Ni foam	260	10	[7]
NiFeMo IOS	Ni foam	198	50	[8]
NiFeMo alloy	Ni foam	238	50	[9]
MoFe:Ni(OOH)/$Ni(OH)_2$	Ni foam	240	47	[10]
NiFe-MoOx	Ni foam	276	55	[11]
Ni_2Fe_1-Mo	Ni foam	231	60	[11]

Table S4. Comparison of NiFeMoN photoanode with recent reported photoanode performance in 1 M KOH of electrolyte.

Catalysts	Substrate	ABPE (%)	Stability (h)	Reference
**NiFeMoN**	**Si**	**5.2**	**76**	**This work**
a-NiFeMo	$BiVO_4$	1.21	0.33	[12]
Ni/NiOOH/NiFe	Si	1.42	20	[13]
NiFeLDH/NiO_x/Ni	Si	4.3	68	[14]
NiFe	Si	3.5	14	[15]
Ni/TiO_2	Si	5.8	200	[16]
AlO_x/Au/Ni/$NiFeO_x$	Si	3.71	80	[17]
Al_2O_3/Ni/NiO_x/NiOOH	Si	3	80	[18]
Ni/NiFe	Si	4.8	10	[19]
TiO2/In2O3	Si	2.25	5	[20]
Ni/TiO_2	Si	4	20	[21]
Ni/TiO_2	Si	3.91	80	[22]

References

[1] S. Anantharaj, K. Karthick, S. Kundu, *Materials Today Energy* **2017**, 6, 1.

[2] W. Zeng, C. Wei, K. Zeng, X. Cao, M. H. Rümmeli, R. Yang, *ChemElectroChem* **2021**, 8, 524.

[3] B. C. Moon, W. H. Choi, K.-H. Kim, D. G. Park, J. W. Choi, J. K. Kang, *Small* **2019**, 15, 1804764.

[4] X. Su, Q. Sun, J. Bai, Z. Wang, C. Zhao, *Electrochimica Acta* **2018**, 260, 477.

[5] Z. Yu, T. Lin, C. Zhu, J. Li, X. Luo, *ChemElectroChem* **2021**, 8, 1316.

[6] M. Baek, G.-W. Kim, T. Park, K. Yong, *Small* **2019**, 15, 1905501.

[7] J. Ekspong, C. Larsen, J. Stenberg, W. L. Kwong, J. Wang, J. Zhang, E. M. J. Johansson, J. Messinger, L. Edman, T. Wågberg, *ACS Sustainable Chemistry & Engineering* **2021**, 9, 14070.

[8] C.-T. Hsieh, C.-L. Huang, Y.-A. Chen, S.-Y. Lu, *Applied Catalysis B: Environmental* **2020**, 267, 118376.

[9] F. Qin, Z. Zhao, M. K. Alam, Y. Ni, F. Robles-Hernandez, L. Yu, S. Chen, Z. Ren, Z. Wang, J. Bao, *ACS Energy Letters* **2018**, 3, 546.

[10] Y. Jin, S. Huang, X. Yue, H. Du, P. K. Shen, *ACS Catalysis* **2018**, 8, 2359.

[11] C. Xie, Y. Wang, K. Hu, L. Tao, X. Huang, J. Huo, S. Wang, *Journal of Materials Chemistry A* **2017**, 5, 87.

[12] Y. Gao, Z. Tian, H. Zhu, H. Xue, L. Ma, Y. Dai, W. Zhao, X. Li, N. Li, L. Ge, *ACS Applied Energy Materials* **2021**, 4, 14649.

[13] Q. Cai, W. Hong, C. Jian, W. Liu, *Nanoscale* **2020**, 12, 7550.

[14] B. Guo, A. Batool, G. Xie, R. Boddula, L. Tian, S. U. Jan, J. R. Gong, *Nano Letters* **2018**, 18, 1516.

[15] Z. Liu, C. Li, Y. Xiao, F. Wang, Q. Yu, M. B. Faheem, T. Zhou, Y. Li, *The Journal of Physical Chemistry C* **2020**, 124, 2844.

[16] B. Liu, S. Wang, S. Feng, H. Li, L. Yang, T. Wang, J. Gong, *Advanced Functional Materials* **2021**, 31, 2007222.

[17] J. Ma, H. Chi, A. Wang, P. Wang, H. Jing, T. Yao, C. Li, *Journal of the American Chemical Society* **2022**, 144, 17540.

[18] Z. Luo, B. Liu, H. Li, X. Chang, W. Zhu, T. Wang, J. Gong, *Small Methods* **2019**, 3, 1900212.

[19] C.-H. Chuang, P.-H. Kang, Y.-Y. Lai, C.-H. Hou, Y.-J. Cheng, *ACS Applied Energy Materials* **2022**, 5, 8483.

[20] G. Yan, Y. Dong, T. Wu, S. Xing, X. Wang, *ACS Applied Materials & Interfaces* **2021**, 13, 52912.

[21] B. Liu, S. Feng, L. Yang, C. Li, Z. Luo, T. Wang, J. Gong, *Energy & Environmental Science* **2020**, 13, 221.

[22] S. Cao, Z. Zhang, Q. Liao, Z. Kang, Y. Zhang, *Energy Technology* **2021**, 9, 2000819.
